# Livestock faecal indicators for animal management, penning, foddering and dung use in early agricultural built environments in the Konya Plain, Central Anatolia

**DOI:** 10.1007/s12520-019-00988-0

**Published:** 2020-01-18

**Authors:** Marta Portillo, Aroa García-Suárez, Wendy Matthews

**Affiliations:** 1 Department of Archaeology and Anthropology, Archaeology of Social Dynamics (2017SGR 995), Institució Milà i Fontanals, Spanish National Research Council (CSIC), Barcelona, Spain; 2 Department of Archaeology, School of Archaeology, Geography and Environmental Sciences, University of Reading, Reading, UK; 3 Department of Prehistory, Autonomous University of Barcelona, Bellaterra, Spain; 4 Oriental Institute, University of Oxford, Oxford, UK

**Keywords:** Neolithic, Central Anatolia, Husbandry, Coprolite, Biomarker, Geoarchaeology

## Abstract

Livestock dung is a valuable material for reconstructing human and animal inter-relations and activity within open areas and built environments. This paper examines the identification and multi-disciplinary analysis of dung remains from three neighbouring sites in the Konya Plain of Central Anatolia, Turkey: Boncuklu (ninth–eighth millennium cal BC), the Çatalhöyük East Mound (eighth–sixth millennium cal BC), and the Late Neolithic occupation at the Pınarbaşı rockshelter (seventh millennium cal BC). It presents and evaluates data on animal management strategies and husbandry practices through the simultaneous examination of plant and faecal microfossils and biomarkers with thin-section micromorphology and integrated phytolith, dung spherulite, and biomolecular analyses, together with comparative reference geo-ethnoarchaeological assemblages. Herbivore dung and other coprogenic materials have been identified predominantly in open areas, pens and midden deposits through micromorphology and the chemical signatures of their depositional contexts and composition. Accumulations of herbivore faecal material and burnt remains containing calcitic spherulites and phytoliths have provided new information on animal diet, fodder and dung fuel. Evidence from phytoliths from *in situ* penning deposits at early Neolithic Çatalhöyük have provided new insights into foddering/grazing practices by identifying highly variable herbivorous regimes including both dicotyledonous and grass-based diets. This review illustrates the variability of dung deposits within early agricultural settlements and their potential for tracing continuity and change in ecological diversity, herd management strategies and foddering, health, energy and dung use, as well as the complexity of interactions between people and animals in this key region during the early Holocene.

## Introduction

There is a growing recognition of the value of animal dung for reconstructing ecological and socio-cultural practices in archaeology. Livestock dung materials found within built environments and open settlement areas bear important information regarding environment, herd management and ecology, foddering and subsistence strategies, energy and fuel use, interactions between people and their animals, health and risk of zoonotic diseases, and socio-economic relations and cultural practices more widely. Livestock penning practices often provide a regular and predictable supply of animal products, including dung, that can be subsequently used in multiple ways, such as fuel, fertilizer—either in its organic form or after being burned—and as temper in building materials (e.g. Kramer 1982; Miller 1984; Anderson and Ertuğ-Yaras 1998; Reddy 1999; Sillar 2000; Shahack-Gross et al. 2004; Milek 2012; Bogaard et al. 2013). The use of dung and a varied range of secondary herd products marks a broader exploitation of resources derived from livestock. The contribution of this archaeological resource since at least the domestication of animals and its role in the so-called ‘secondary products revolution’ (Sherratt 1983), however, have not been fully evaluated from a local perspective in light of the emergence and spread of farming.

The accurate identification of dung remains in the field is not straightforward and it frequently requires laboratory-based analytical techniques, such as calcitic spherulite studies and biomolecular analyses of sterols and bile acids. This is mostly due to the many different forms in which archaeological dung occurs, for instance as charred aggregates, trampled layers in pens, and dung ashes and disaggregated inclusions in construction materials, which demands a multi-disciplinary approach in order to meet the challenges associated with detecting these materials. In addition, methodological issues related to the use of routine sampling techniques such as sieving and flotation result in faecal materials and their contents being irreversibly disaggregated or mixed with other organic and inorganic components of different origin. Over the last three decades a variety of geoarchaeological, archaeobotanical, biochemical, as well as comparative ethnoarchaeological and experimental methodological approaches have enabled the identification and interpretation of dung in the archaeological record (Shahack-Gross 2011, and references therein). Among these multiple and diverse lines of evidence, the strength of high-resolution integrated micro-contextual approaches to animal faecal matter lies in their ability to examine its contents and signatures within its depositional and socio-economic contexts. These methods contribute to understanding the depositional pathways and taphonomic histories of dung, critical for the identification of activity areas and practices related to animal management (e.g. penning, dung burning, discarding/dumping, and construction/plastering) and their archaeological implications for site formation processes, concepts and uses of space within a settlement, and health. Comparative geo-ethnoarchaeological and experimental research on dung assemblages provides an important framework for examining the significance of dung indicators and their contextual associations, as well as the ecological and anthropogenic factors influencing these (e.g. Brochier et al. 1992; Canti 1999; Shahack-Gross et al. 2003, 2004; Macphail et al. 2004; Milek 2012; Portillo et al. 2014, 2017; Elliott et al. 2015; Friesem 2016; Prost et al. 2017; Égüez et al. 2018).

**Fig. 1:**
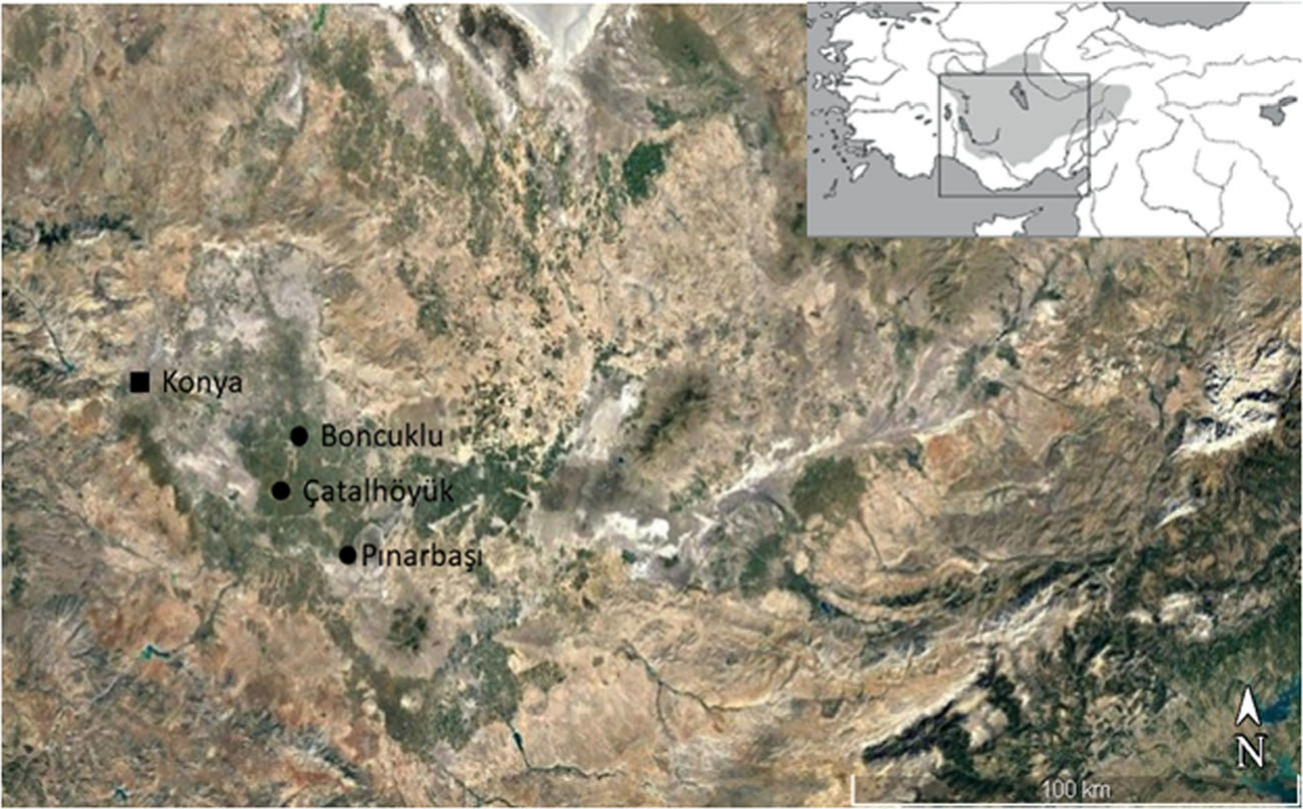
Map of the Konya Plain in Central Anatolia (Turkey) showing the archaeological sites mentioned in the text

This review presents and evaluates interdisciplinary data on research conducted on the dung of livestock found within early Holocene built environments and open spaces from the Konya Plain, an extensive area of south-central Anatolia, Turkey (Fig. 1). The Konya Plain is currently recognized as a region of key importance for understanding the development and spread of Neolithic innovations. These included the adoption of animal domestication and the development of dairying activities and use of livestock for secondary purposes, based on several decades of archaeological and palaeoenvironmental research, including field survey, high-resolution coring, excavations and extensive spatial sampling of early settlements (e.g. Baird 1996, 2005; Baird et al. 2012, 2018; Martin et al. 2002; Russell et al. 2005; Evershed et al. 2008; Bogaard et al. 2017; Roffet-Salque et al. 2018). This review focuses on a selection of sites in this core region in which a range of dung deposits have been found: the early agricultural settlement of Boncuklu (8300–7800 cal BC), the Çatalhöyük East Mound (7100–5950 cal BC) and the Late Neolithic occupation of the Pınarbaşı rockshelter (6500–6000 cal BC) (e.g. Matthews et al. 1996; Matthews 2005a, b; Shillito et al. 2011; García-Suárez 2017; García-Suárez et al. 2018). It aims to evaluate the contribution of interdisciplinary sampling strategies and analytical advances to the development of high-resolution studies of livestock dung using integrated micromorphological, phytolith and dung spherulite analyses, and organic biomarker analyses, coupled with comparative ethnoarchaeological references from the Central Anatolian plateau (Anderson and Ertuğ-Yaras 1998; Yalman 2005; Portillo and Matthews accepted). This paper further highlights the value of interdisciplinary analytical methods in geoarchaeology, archaeobotany and biochemistry and comparative geo-ethnoarchaeological data enabling robust identification and interpretation of dung assemblages and their archaeological significance for exploring the emergence and developments of early farming life-ways.

This paper, therefore, provides a timely review of the development of integrated high-resolution approaches to the study of animal dung. This review begins with an introduction to the regional environment, modern husbandry, grazing and dung use, followed by a brief evaluation of the nature of these archaeological sites and early farming. It then summarizes the key methodologies and techniques used in the study of modern and archaeological dung in the study area, and reviews their potential applications, abilities and challenges, and contribution to interdisciplinary studies of faecal remains. Discussion focuses on current debates on early farming development and resilience, concepts of space and health of early settled life, and the complexity of relationships between people and livestock across territories in Central Anatolia during the early Holocene.

## The Konya plain

### Physical environment

The Konya Basin occupies an extensive inland drainage plateau in south-central Anatolia that extends over 1,000,000 ha with an average altitude of about 1000 m a.s.l. (Kuzucuoğlu et al. 1999). The Konya Plain is located towards the west of the basin and is surrounded by the Taurus Mountains to the south and the Anatolides to the north and west. The Çarşamba alluvial fan covers an area of approximately 474 km^2^ that hosts a number of early Neolithic settlements, including Çatalhöyük and Boncuklu (Roberts et al. 1999) (Fig. 1). The Konya Basin is a closed pluvial basin that has actively responded to changes in climate and precipitation, with a highly variable landscape in time and space (Ayala et al. 2017). The results from high-resolution coring conducted since 2007 indicate increased wetness during the last stages of the Pleistocene and the early Holocene with localized pockets of wetter conditions, interpreted as characteristic of a humid anabranching channel system. During the Neolithic occupation on the Çatalhöyük East Mound the fluvial regime shifted from humid to dryland anabranching conditions, and infilling of the undulating topography of the eroded marl of the former Pleistocene palaeolake continued (Ayala et al. 2017). This model differs from previous palaeoenvironmental reconstructions that argued that the site was ‘largely surrounded by backswamp-type soils’ and that its vicinity was subject to seasonal flooding conditions (Roberts et al. 1996, 1999, 2007; Boyer et al. 2006).

The present-day climate is dominated by cold semi-arid/steppe conditions, characterized by hot, dry summers and cold, wet winters. The average annual precipitation is 350 mm and the area experiences considerable seasonal temperature fluctuations of over 20 °C between the warmest and coolest months, including a period of drought between July and September (Fontugne et al. 1999; Ayala et al. 2017). This climate regime currently gives rise to predominantly semi-arid steppe vegetation that has also been impacted by human settlement and agro-pastoral production, especially by long-term grazing pressure. Traditionally, pastoral grazing of sheep and cattle has been fundamental to the livelihoods of local communities, a practice that has led to heavy grazing disturbance on rangeland vegetation over the last three decades (Fırıncıoğlu et al. 2007).

### The archaeological sites and early husbandry

The settlement mound of Boncuklu, with an occupation spanning *c*. 8300–7800 cal BC, has provided evidence of the early appearance of agriculture in the Konya Plain during the second half of the ninth millennium cal BC (Baird et al. 2012, 2013, 2018). The site extends over approximately 1 ha and includes sub-oval domestic buildings with mudbrick superstructures surrounded by open spaces where midden deposits accumulated. The later settlement mound at Çatalhöyük (7100–5950 cal BC) (Bayliss et al. 2015), is > 13 ha in size with extensive mudbrick architecture and middens/open areas. The site has been the focus of pivotal debates regarding Neolithic innovations, agricultural practices defined broadly here as plant cultivation and animal herding, and responses and resilience to environmental change in the early Holocene (Hodder 2006, 2007, 2013; Ayala et al. 2017; Bogaard et al. 2017). The Late Neolithic occupation at the Pınarbaşı rockshelter (*c*. 6500–6000 cal BC), with a maximum extent of 400 m^2^, displays sub-oval built environments with wattle and daub superstructures and multiple evidence of pastoral activities, including a high proportion of herded sheep in the faunal assemblage, which comprises abundant foetal and neonate remains (Baird et al. 2011).

The Konya Plain was home to contemporary sedentarising communities in the late ninth and early eighth millennium cal BC with quite distinctive cultural identities and contrasting economic strategies (Baird 2012; Baird et al. 2018). The Boncuklu community practiced low-level crop cultivation with sheep and goat faunal remains occurring in very low proportions with an uncertain wild/domestic status based on bone morphometrics (Baird et al. 2012, 2018). By contrast, wild caprines are present in high numbers at ninth millennium Pınarbaşı. There is evidence for a more substantial mixed-farming economy at the contemporary central Anatolian site of Aşıklı Hüyük, in Cappadocia, with a range of crops and the occurrence of caprine management by 8200 cal BC (van der Zeist and Roller 1995, 2003; Özbaşaran 2012; Stiner et al. 2014; Abell et al. 2019). Large-scale mixed farming including both the cultivation and management of fully domestic cereals, legumes, and caprines are attested by at least 7100 cal BC on the Konya Plain at Çatalhöyük, followed by the adoption of domesticated cattle (*Bos taurus*) and use of secondary animal products such as milk in the Late Neolithic (Russell et al. 2005, 2013; Evershed et al. 2008).

## Methodology

### Ethnoarchaeology and modern livestock dung

**Fig. 2:**
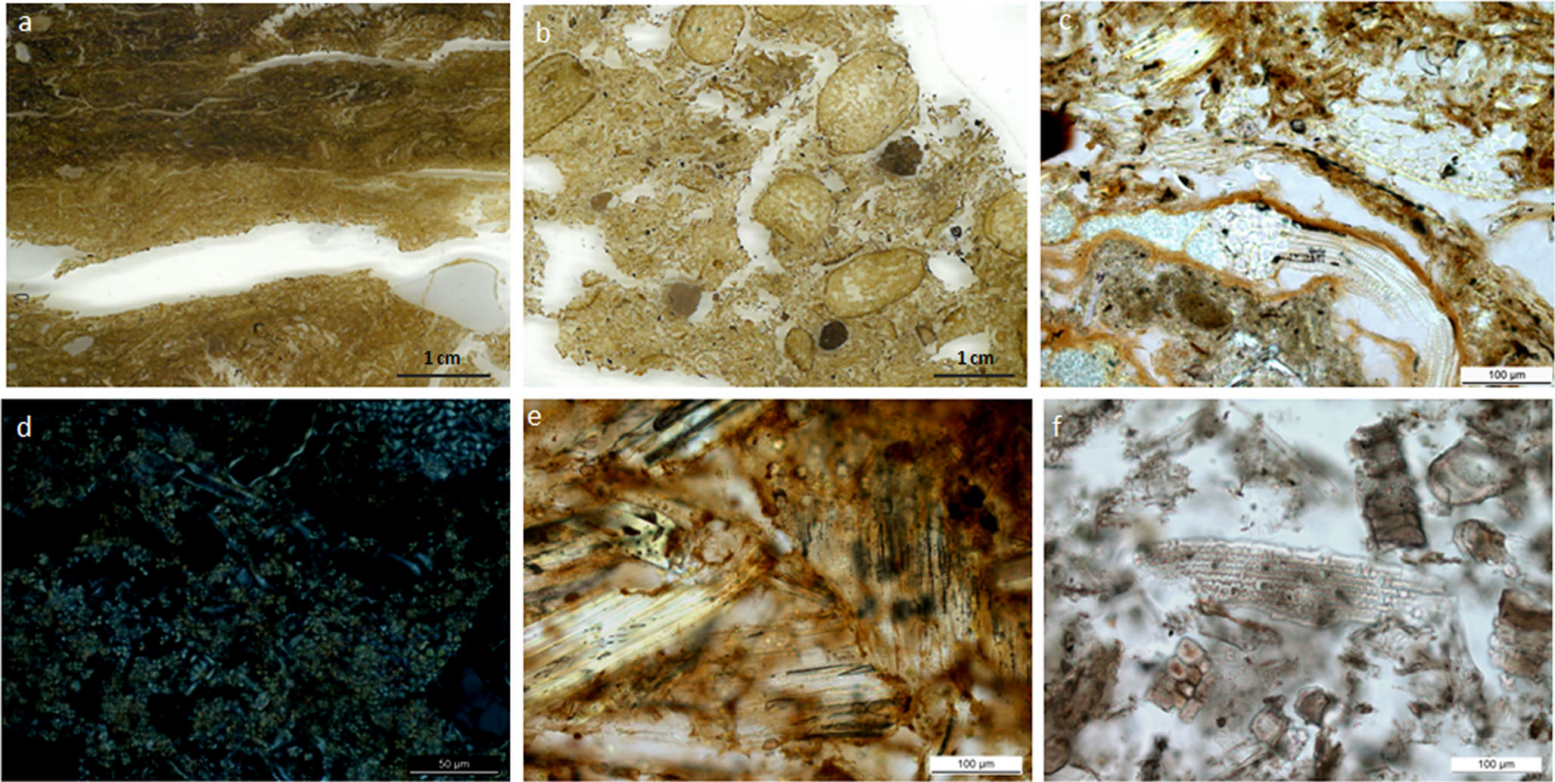
Modern dung (*tezek*) cakes and fuel ashes from Central Anatolia collected by Anderson and Ertuğ-Yaras (1998). **a** Scan of a resin-impregnated thin section showing the macroscopic laminar structure of compacted trampled cattle dung within an enclosure, dominated by organic matter but containing mineral inclusions; **b** scan of a thin section of a dung cake including intact sheep faecal pellets and mineral inclusions; **c** detail of a sheep dung cake manufactured in winter showing multi-celled or anatomically connected phytoliths of dicotyledonous leaf tissues, PPL; **d** detail of the same sheep dung cake (image c) composed of calcitic spherulite-rich assemblages, XPL; **e** detail of a cattle dung cake displaying multicelled or anatomically connected phytoliths from the leaves and culms of grasses, PPL; **f** grass-dominated phytolith assemblages from cattle dung fuel ashes, PPL

Ethnographic surveys of modern husbandry practices and ethnobotanical studies were conducted during the 1990s in two rural villages: Pınarbaşı, a small settlement located on the edge of the Taurus Mountains in the Karaman district, and Kizilkaya, a larger village on the Melendiz Plain, in Aksaray district (Ertuğ-Yaras 1996, 1997; Anderson and Ertuğ-Yaras 1998). The particular focus of the later work was the examination of foddering practices, animal dung or *tezek* (the generic Turkish name for dung) and the manufacture of dung cakes, fuel storage and burning, and choices and taboos regarding the use of dung as fuel, as well as the study of the macro-botanical composition of fodder and dung materials (mostly from sheep and cattle). The manufacture of dung cakes made from mixed cow and sheep pellets was further reported in ethnoarchaeological research in the small village of Türkmencamili, in the Çumra Plain, in the Konya province (Yalman 2005). Anderson and Ertuğ-Yaras recorded different types of dung cakes, mainly manufactured during spring and summer: unprocessed dung or droppings collected from the field or enclosures, trampled dung dug out from pens, cattle dung compacted and moulded by hand into rounds (*yapma*), and cattle dung compressed into a wooden mould (*kepiç*). A variety of firing installations were reported including the so-called *tandir*, a ventilated underground oven similar to Near Eastern tannurs used for cooking and fuelled with *tezek* cakes. Comparative integrated phytolith and dung spherulite analyses from micromorphological thin-sections of modern dung samples collected by Anderson and Ertuğ-Yaras (1998), have been recently used to provide further information on dung morphology and microfossil dung content in a selection of contexts from all three neighbouring settlements (Fig. 2) (Portillo and Matthews accepted).

### Archaeological dung sampling and analytical methods

Archaeological dung remains have been examined through both spot and block sediment samples for contextual micromorphological and integrated geochemical, mineralogical, bioarchaeological, and biomolecular analyses, and comparative ethnobotanical and geo-ethnoarchaeological modern datasets (Anderson and Ertuğ-Yaras 1998; Portillo and Matthews accepted). Contrary to other sampling procedures such as flotation and sieving in which dung and its contents may be disaggregated, irreversibly mixed or lost, integrated sampling strategies allow the high-resolution examination of undisturbed stratigraphy and the simultaneous study of diverse organic, inorganic and microartefactual components *in situ* within their depositional context (e.g. Matthews et al. 1996; Matthews 2005a). For a review on materials and methods for identification and techniques used in the study of modern and archaeological dung along with their potential applications, research rationale and limitations see Table 1 in Shahack-Gross (2011) and Table 1 in Portillo and Matthews (accepted).

In the field, dung samples were collected by cutting sediment blocks and collecting high-resolution spot samples from stratigraphic sequences suspected to contain faecal remains at the macroscale. Dung was identified where possible in the field laboratory by microscopic examination of related spot samples through the presence of calcitic spherulites displaying a cross of extinction under crossed polarized light. These microfossils form in the digestive system of many animals, particularly ruminants, and can be identified in varying proportions in their faeces (Canti 1999). Calcitic spherulites have been found to be common in modern livestock materials from the study region, including dung cakes and fuel ashes (Portillo and Matthews accepted). Archaeological spot sediment samples were firstly examined under the optical microscope by mounting non-permanent slides with clove oil to provide rapid feedback during excavation on the presence or absence of dung spherulites and other plant microfossils such as phytoliths and ash pseudomorphs (resulting from the burning of wood to at least 450 °C) and for a pilot scanning of their vegetal components (e.g. Matthews 1999, 2005a; Matthews and Portillo 2017). *In situ* penning at Neolithic Çatalhöyük was identified firstly in the field by a distinctive laminar microstructure of trampled herbivore dung with parallel oriented articulated phytoliths, and confirmed in the field laboratory by microscopic spot smear slide identification of abundant spherulites (Matthews and Portillo 2017; Portillo et al. 2019). This high-resolution spot sampling was conducted for suspected coprolitic materials collected during fieldwork at all three sites (e.g. Matthews 2005a; Shillito et al. 2011; García-Suárez et al. 2018). In addition, a method of ‘microsampling’ block sub-samples from thin-section blocks before impregnation enabled direct comparisons between detailed quantitative microfossil contents and micromorphological observations (Matthews et al. 2004; Shillito 2011, 2017; García-Suárez et al. 2018). Further information was then obtained on the defecator’s diet and food regime by the identification of a varied range of faunal and vegetal remains in thin-section and in spot samples. The identification of defecators was investigated through biomolecular analyses by Gas Chromatography Mass Spectroscopy (GC/MS) targeting both sterols and bile acids to discriminate between animal and human coprolitic materials (Bull et al. 2005; Shillito et al. 2011, 2013a, b; Ledger et al. 2019). Further evidence from the analysis of intestinal parasites in coprolites by Ledger et al. (2019) has enabled new insights on the health conditions of the inhabitants of Çatalhöyük.

## Archaeological significance

Dung remains have been found to represent important archaeological materials at Çatalhöyük, particularly in open areas including midden and penning deposits, as identified through the micromorphological observation of their depositional contexts and composition by microfossil and biomarker signatures (Matthews et al. 1996; Matthews 2005a; Rosen 2005; Shillito 2011, 2017; Shillito et al. 2008, 2011; Shillito and Matthews 2013; García-Suárez et al. 2018, accepted-a; Portillo et al. 2019). Faecal remains have been more recently investigated in middens at the earlier settlement of Boncuklu and the Late Neolithic occupation of the Pınarbaşı rockshelter by applying similar integrated micromorphological and microfossil methodological approaches (García-Suárez et al. 2018, accepted-b). Midden deposits in all three sites are characterized by depositional sequences with finely stratified layers and components that are difficult to resolve in the field, making the application of integrated micro-contextual methods highly relevant to the investigation of their complex depositional and post-depositional pathways (Baird et al. 2012; Shillito and Matthews 2013; García-Suárez et al. 2018). In the field and in thin-section, omnivore faecal materials may be identifiable as a distinctive orange/yellow fine material with inclusions such as embedded bone fragments and plant remains such as hackberry pericarps. GC/MS analyses in a range of cases have indicated that some of these are of human, rather than pig or dog, at the sites examined with significant implications for human diet and health and concepts of space for early settled life (Matthews et al. 1996; Matthews 2005a; Shillito et al. 2011; Bull et al. 2005; Shillito et al. 2011, 2013a, b).

To aid in the identification of animal dung and animal management practices, ethnographic research has been conducted in these areas, resulting in comparative samples and reference datasets of livestock dung remains and dung-products from site vicinities in the central Anatolian plateau (Anderson and Ertuğ-Yaras 1998; Yalman 2005; Portillo and Matthews accepted). These integrated approaches to this core region for understanding the origins and spread of agriculture provide an ideal case-study for high-resolution investigations of dung material aimed at exploring continuity and change in animal management strategies and early husbandry.

### Penning and animal management

Recent studies focusing on the analysis of integrated micromorphological and plant and faecal microfossil evidence from dung assemblages in open areas from the study sites have contributed to understanding animal management strategies in this region, providing new insights into early farming in the Konya Plain through time (Baird et al. 2018; García-Suárez et al. 2018). At Boncuklu and Pınarbaşı micro-contextual studies have not identified *in situ* penning locations, although herbivore dung remains have been documented at both sites associated with fire-related activities. Thin-section micromorphology detected direct evidence of *in situ* penning in the early and middle occupation levels at Çatalhöyük (Matthews et al. 1996, 2014; Matthews 2005a; Cessford 2007; Matthews and Portillo 2017; García-Suárez et al. accepted-a; Portillo et al. 2019). These deposits were identified by their distinctive laminar microstructure oriented parallel to the pen surface, caused by physical compaction as a result of intense animal trampling and from the presence of fodder and animal faeces (Macphail et al. 2004; Shahack-Gross 2017; Portillo and Matthews accepted). Observations in micromorphological thin-sections from modern reference ethnoarchaeological materials from herbivorous herds from the research area (Fig. 2a and b) show that penning deposits may include amorphous organic matter and organic phosphate staining, commonly pale to dark brown under plane light (PPL). These commonly include partially digested to undigested plant tissues and seeds, plant microfossils such as phytoliths and calcium oxalates, cellulose displaying interference colours under crossed polarized transmitted light (XPL), and other distinctive dung microfossils such as calcitic spherulites and spores, although their composition may vary significantly according to animal producers and their diet (Anderson and Ertuğ-Yaras 1998; Portillo and Matthews accepted).

At Çatalhöyük, the identification of penning areas distributed in the South and North Areas of the East mound provides direct evidence of animal management within the settlement (Matthews et al. 1996; Matthews 2005a; Cessford 2007; Matthews and Portillo 2017; García-Suárez et al. accepted-a; Portillo et al. 2019). In the early occupation of the South Area, two penning areas were identified, the first one in the deep sounding excavated in the late 1990s, and the second one during the last field season of the Stanford project in 2017 (Fig. 3 in Matthews and Portillo 2017). In the North Area, a penning sequence was also discovered in this last field season (Matthews and Portillo 2017; García-Suárez et al. accepted-a; Portillo et al. 2019). These new penning areas provide direct evidence for the management of herds in enclosures within the boundaries of the site immediately prior to a period of settlement expansion in the South Area, and increased exploitation of the wider landscape (Pearson et al. 2007, 2015; Pearson 2013; Russell et al. 2013; Larsen et al. 2013; Matthews 2018; Middleton 2018). Penning deposits have also been identified within buildings, such as in Space 470 in the South Area, although these are generally less substantial and more short-lived than those observed in open areas. Here, superimposed thin layers of trampled herbivore dung observed through micromophology attest the occasional use of this built environment as a pen, possibly temporarily to host birthing or sick animals, given the low amounts of faecal matter observed in thin-section (Barański et al. 2015). This overall patterning suggests that from *c*. 7000–6700 BC some animals were penned within the site of Çatalhöyük. Isotopic analyses of animal bone indicate that later in the Neolithic occupation, coincident with an increased population size and greater mobility for herding and acquiring food and other resources, animals were kept further away from the site (Pearson et al. 2007; Henton 2012, 2013; Larsen et al. 2019). Significantly, this isotopic evidence pointing to the herding of animals at greater distances from the site corresponds with a decrease in the amount of dung that was discarded in open areas, as the micromorphological evidence from the final levels of occupation in the East Mound suggests (García-Suárez et al. 2018).

**Fig. 3:**
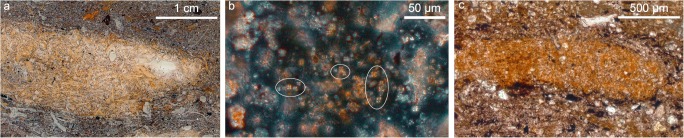
Dung occurrences in multiple contexts at the study sites: **a** detailed view of micromorphological thin-section 367 comprising omnivore coprolite accumulations in an open area at Boncuklu; **b** photomicrograph of calcitic spherulites in a charred herbivore faecal aggregate embedded in dung ashes at the Pınarbaşı rockshelter, XPL (distinguishable spherulites are circled); **c** trampled charred faecal aggregate embedded in discarded fuel materials within hearth/oven rake-out context in Building 114 at Çatalhöyük, PPL

The identification of animal penning has important implications for studies on continuity and change in animal management patterns, site organization and concepts of space, as well as on early settled life conditions and health challenges associated with early farming and food production (Portillo et al. 2019). Futher, the presence of pens for domestic animals and refuse areas cointaining animal dung and human coprolites in close proximity to crowded living spaces bears critical implications regarding hygiene, health and well-being in the community and an increased exposure to pathogenic microorganisms including parasites, bacteria, and viruses and the potential spread of diseases within the settlement (Larsen et al. 2019). The parasites identified in two human coprolites to date from Çatalhöyük, however, are geohelminths that spread from human to human rather than from animals to humans (Ledger et al. 2019). These coprolites are from the late levels of the site from miden deposits in Space 329, Level South P, dating to *c*. 6410–6150 BC (Ledger et al. 2019), a time when isotopic evidence suggests that animals were grazed and kept further away from the settlement, as discussed above (Pearson et al. 2007; Henton 2012, 2013). Further analyses of coprolites may reveal evidence of zoonotic parasites that spread from animals to humans, as commonly identified on Neolithic sites in the Mediterranean (Anastasiou and Mitchell 2013) and other traces of disease. The presence of animal pens within the settlement from at least *c*. 7000–6700 BC, discussed here, suggests that there would have been greater risk of zoonotic diseases during these earlier periods of occupation when humans and animals lived in greater proximity.

### Animal diet, foddering/grazing, seasonality and ecological diversity

The nature of livestock diet varies according to a range of factors including ecological and seasonal variability in food sources, sex/age-based dietary requirements, selection of feed by animals and humans, and management practices, as illustrated by ethnographic and ethnobotanical research in the study area (Anderson and Ertuğ-Yaras 1998). Most of the reported archaeological information on animal diet comes from microfossil evidence such as phytoliths in thin-section, which enables observations of dung materials and their components *in situ* in an undisturbed form, rather than macrobotanical remains that are commonly disaggregated or mixed through flotation or sieving. Whilst dietary practices have been investigated by isotopic signatures of crops and both animal bone and teeth from several sites in the Konya Plain (Pearson et al. 2007, 2015; Henton 2012; Pearson 2013; Bogaard et al. 2013; Wallace et al. 2015; Middleton 2018; Baird et al. 2018), isotopic analyses of faeces are currently an under-developed area of study and a promising field for future research (e.g. Codron et al. 2005; Shahack-Gross 2011).

**Fig. 4:**
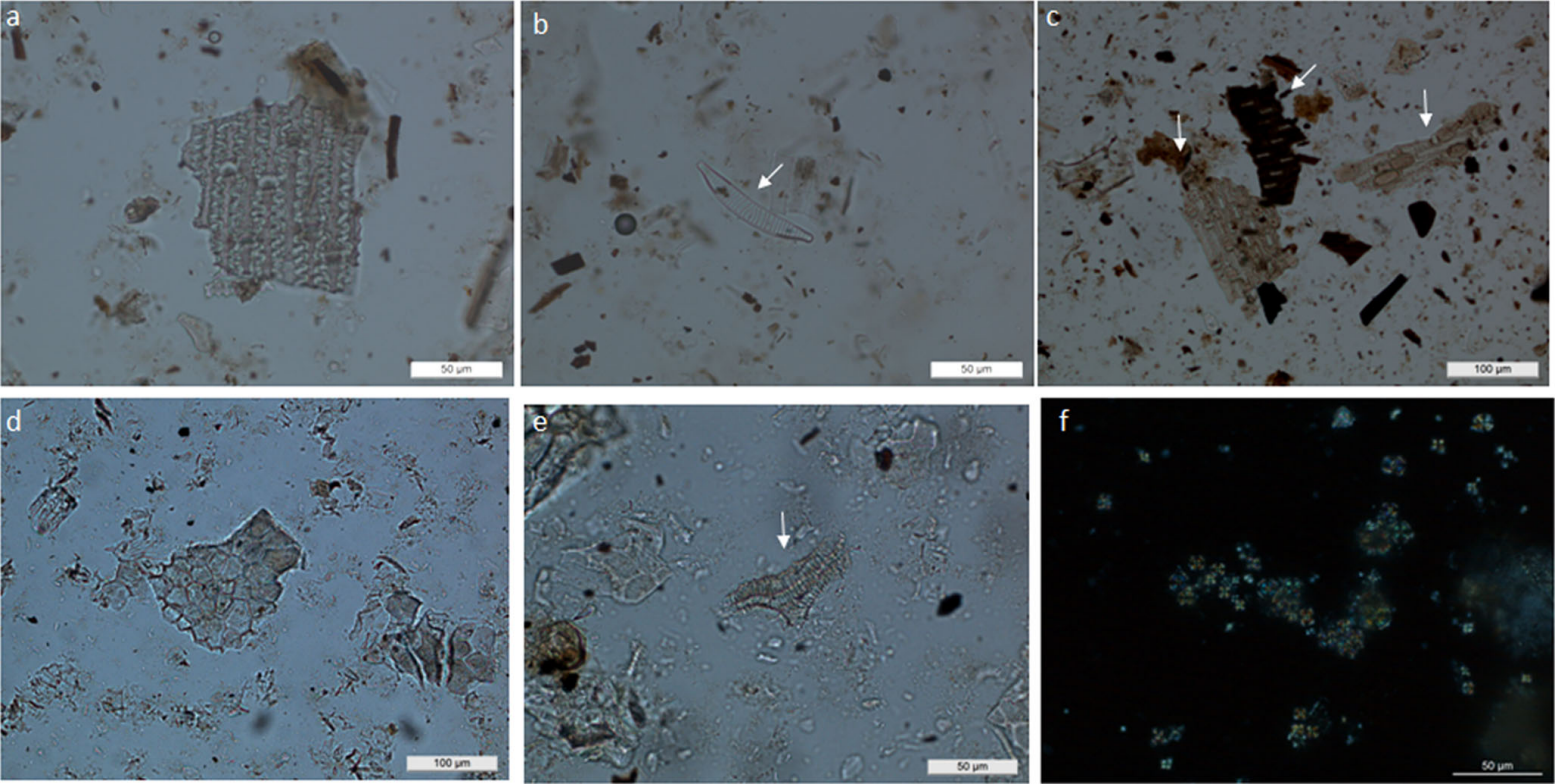
Photomicrographs of phytoliths and other microfossils identified in archaeological samples from Boncuklu (BK, Area M), Pınarbaşı (PB, Area B) and Çatalhöyük (CH, South Area) (400× or 200×). **a** Multi-celled or anatomically connected phytoliths derived from the husks of grasses from omnivore faecal contexts (BK); **b** diatom from faecal contexts (BK); **c** multicelled or anatomically connected phytoliths from the leaves and culms of reeds and Pooideae grasses from burnt-herbivore dung deposit (PB); **d** dicotyledonous epidermal leaf tissues from penning deposits (CH); **e** tracheid phytoliths from dicotyledonous leaves from penning deposits (CH); **f** clusters of calcitic dung spherulites from penning deposits (CH)

At Boncuklu, faecal remains from midden deposits (Fig. 3a) display very low spherulite content in thin-section, and highly variable phytolith types and abundance (García-Suárez et al. 2018). These mainly derive from Pooideae grasses and, to a lesser extent, from reeds and sedges, which are common in wetland environments; few assemblages are particularly rich in multicelled phytoliths from the husks of these plants (Fig. 4a). Some of these coprolites also contain abundant digested bone remains, with morphologies suggesting a microfaunal origin, possibly birds and amphibians. Preliminary GC/MS results point towards a possible human origin for these faecal materials (I. Bull, pers. comm.; García-Suárez et al. 2018). Additional biogenic microfossils such as diatoms and, to a lesser extent, sponge spicules were also common in many of the phytolith slides (Fig. 4b), and may potentially serve as indicators of well-watered environmental conditions (Wilding and Drees 1971; Schwandes and Collins 1994; Coil et al. 2003). Diatom silica frustules are found in water and in conditions where moisture is present, including deposits and soils, and can also be found in animal faeces resulting from drinking water and ingested matter, as illustrated in geo-ethnoarchaeological studies (Brochier et al. 1992; Portillo and Albert 2011; Portillo et al. 2012; Portillo and Matthews accepted). Although very few traces of herbivore dung have been identified to date at Boncuklu in comparison to omnivore coprolitic remains, the micromorphological identification of charred dung pellets in hearth fills has significant implications for the study of early animal management strategies at this site (García-Suárez et al. 2018, accepted-b). In support of this, although caprines only comprise 4.9% of the identified animal species at Boncuklu (Baird et al. 2012, 2018; Middleton 2018), stable isotope data show a high δ^15^N value in some caprine bones from the site, similar to the later caprines from Çatalhöyük, which appears to indicate an increased consumption of plants growing in the more arid, saline marsh areas of the plain, rather than vegetation from the natural habitat of caprines in the surrounding hills. This has been interpreted as the result of human management and small-scale experimentation with caprine herding in the vicinity of the site (Baird et al. 2018), and is supported by the micromorphological identification of dung from animals that are likely to have been proximate to the site.

**Fig. 5:**
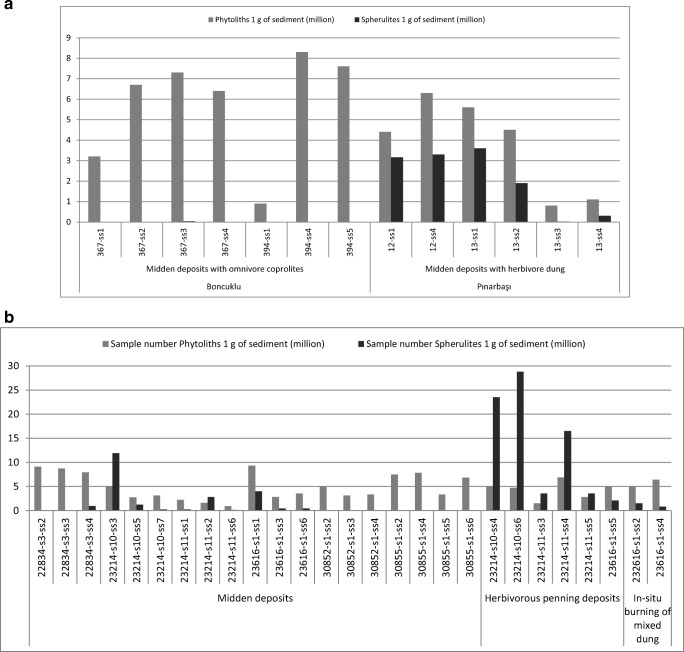
Plot showing absolute concentrations of phytoliths *vs*. dung spherulites obtained from micromorphological block sub-samples. **a** Boncuklu (Area M) midden deposits with abundant omnivore coprolite inclusions and Pınarbaşı (Area B) late middens composed of herbivore dung; **b** Çatalhöyük midden and penning deposits from different locations (South Area, North Area, GDN and TPC). Sample extraction and quantitative analyses followed the methods of Katz et al. (2010) and Canti (1999), respectively. For sample description, contextual field records, and full quantitative data see García-Suárez et al. 2018 and Portillo et al. 2019

In contrast to Boncuklu, numerous faecal deposits characteristic of herbivores have been identified in later middens at seventh millennium Pınarbaşı, with abundant phytolith and calcitic spherulite assemblages (Figs. 4c and 5a). These faecal remains are likely derived from ovicaprines, as the faunal record includes a substantial proportion of herded sheep (Baird et al. 2011). Of particular interest to animal management practices, the zooarchaeological remains included sheep foetuses and neonates from spring birthing, suggesting at least a seasonal presence of herds during the Late Neolithic occupation of the site. The micromorphological observations are predominantly from dung burnt as fuel, and indicate that this was a readily available energy supply, concomitant with the presence of these herds. The burnt fuel deposits include abundant dung spherulites and phytoliths from wild grasses, and leaves and culms of reeds suggested to derive from fodder and fuel sources (García-Suárez et al. 2018).

Direct evidence for herd foddering/grazing practices at Çatalhöyük comes from the *in situ* compacted dung deposits from penning discussed above (Matthews et al. 1996; Matthews 2005a; Cessford 2007; Matthews and Portillo 2017; García-Suárez et al. accepted-a; Portillo et al. 2019). Integrated micromorphological and microfossil analyses suggest that there was significant variation in animal diet, including both grass-based and dicotyledonous diets (Matthews and Portillo 2017; Portillo et al. accepted). A diet enriched in dicotyledonous matter may suggest human management of herd fodder, or it may also relate to possible selection by obligate browsers, such as caprines and, possibly may reflect some degree of seasonality (Rasmussen 1993; Macphail et al. 1997*;* Halstead and Tierney 1998*;* Rosen 2005; Tsartsidou et al. 2008). Phytolith-rich assemblages from dicotyledonous epidermal leaf tissues have been also observed in modern sheep dung cakes manufactured in winter pointing to a diet that is either based on or includes a component of dicotyledonous fodder (Fig. 2c) (Portillo and Matthews accepted). These findings reveal highly variable diets among herbivorous foddering/grazing practices within the boundaries of the early occupation of the site, although with an important input of grasses and reeds through the settlement lifetime.

### Dung burning and the use of dung in construction materials

**Fig. 6:**
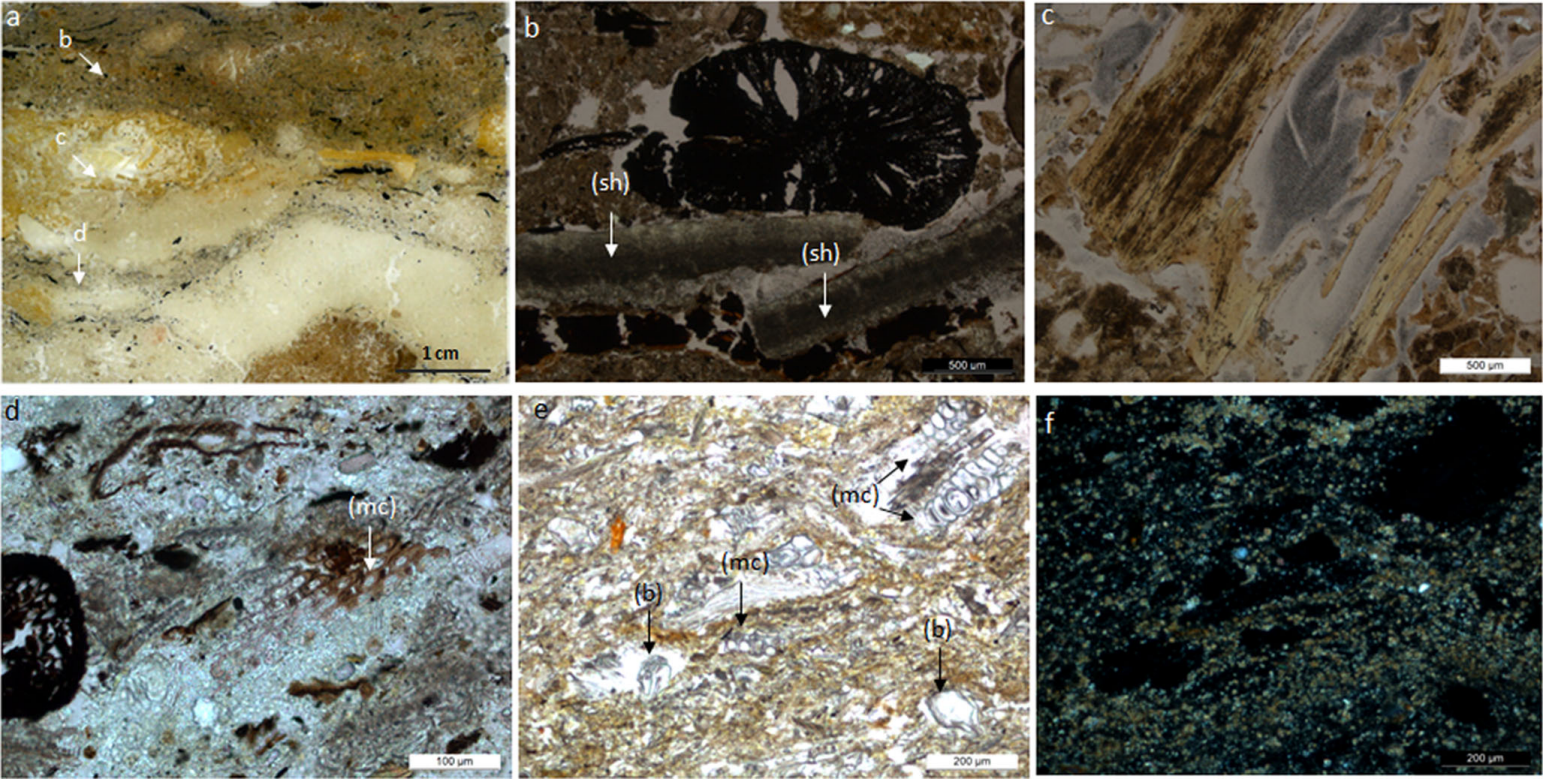
Photomicrographs from thin sections from the Çatalhöyük deep sounding sequence excavated in the late 1990s in the South Area. For sample provenance and micromorphological descriptions see Matthews (2005a, b). **a** Scan of a resin-impregnated thin section showing the location of photomicrographs from Space 181 midden sequence (unit 5290), including organic deposits (**b**), coprolitic materials (**c**) and rake-out (**d**); **b** organic-rich midden deposits with charred plant material and shells (sh), taken at 40× under PPL; **c** omnivorous coprolite containing abundant bone inclusions embedded within amorphous orange/yellow organic matter, PPL; **d** thick calcitic ashy layers (~ 3–1 mm) rich in charred plant remains, multi-celled phytoliths with stomata (mc) from reeds, and calcitic spherulites indicative of dung ashy rake-out, PPL; **e** penning sequence formed by compacted dung deposits from unit 4710 particularly rich in articulated or multicelled (mc) bulliform phytoliths (b) and epidermal tissues from the leaves and stems of reeds oriented sub-parallel, and dung spherulites, indicative of herbivorous grass-rich diets, PPL; **f** the same view of the penning deposit under XPL

Fire-installations such as hearths and ovens fuelled with a range of materials including animal dung have long played a vital role in many societies creating living places and impacting landscapes and life-ways (e.g. Matthews 2016, and references therein). In the Central Anatolian plateau, dung (*tezek*) has long been used as a fuel resource, either in its air-dried organic form as unprocessed droppings, as compacted dung deposits dug out from enclosures, or through the manufacture of dung cakes as outlined below (Anderson and Ertuğ-Yaras 1998; Portillo and Matthews accepted). When dung is burnt as fuel, faecal materials may also be distributed within the built environment as rake-out adjacent to fire installations and in middens with discarded hearth/oven rake-out. Dung material of suspected herbivore origin has been identified in the form of charred and partially calcined aggregates with abundant burnt plant materials in accumulated deposits from all the study sites. Contexts include open areas/middens and buildings, comprising *in situ* fuel in hearths and ovens, and adjacent rake-outs or refuse deposits when buildings are abandoned (Fig. 6a). Dung fuel has been widely identified at Çatalhöyük in micromorphological thin-sections and is also attested in macro-botanical charred plant assemblages through the occurrence of charred dung pellets as well as the presence of wild seeds commonly consumed by animals (Matthews 2005a; Rosen 2005; Fairbairn et al. 2005; Ryan 2011; Shillito 2011; Bogaard et al. 2013, 2014). Wood fuel was also widely used at Çatalhöyük (Asouti 2005; Kabukcu 2018). Integrated archaeobotanical and micromorphological samples of *in situ* fire installations have concluded that fuel sources comprising dung and plant resources such as wood and reeds/grasses were frequently mixed, a pattern that seems common at all the study sites through time.

In thin-section, burnt faecal aggregates generally vary in colour (PPL) from yellowish, dark brown, to pale greyish brown or greyish white, depending on burning temperatures (Fig. 3c). At Çatalhöyük, plant inclusions embedded in herbivore dung remains often include abundant phytoliths, some of which may show evidence of partial melting, indicating the potential of dung fuel to reach high temperatures exceeding *c*. 750–850 °C (Canti 2003). At this site, partially melted phytoliths derive from a range of plants including reeds, sedges and Pooid grasses, as the morphologies of most original cells were preserved and, in most case-studies, identifiable (e.g. García-Suárez et al. 2018; Portillo et al. 2019). The partial melting of phytoliths is also noted in modern reference samples of dung fuel ashes, resulting from the burning of dung cakes composed of grass-dominated assemblages. Further, partially melted phytoliths have been identified in association with darkened dung spherulites, a frequent alteration of these microfossils when exposed to temperatures between 600 and 700 °C (Canti and Nicosia 2018). Evidence of these high firing temperatures has provided insights into a range of fire-related activities at Çatalhöyük, including cooking and lime-burning (Matthews 2005a; Matthews et al. 2013; García-Suárez et al. 2018, accepted-a; Portillo et al. 2019). High burning temperatures can be reached rapidly in closed or walled firing installations such as ovens when well ventilated. Temperatures as high as 850 °C were reached within a few minutes after lighting in modern traditional mud ovens operated with dung-dominated fuels, as reported in geo-ethnoarchaeological studies from different geographical regions (Gur-Arieh et al. 2013; Portillo et al. 2014, 2017). Dung fuels can sustain temperatures for longer than wood sources, remaining hot several hours after the end of the activity (e.g. cooking, baking, and boiling; for pottery manufacturing see e.g. Sillar 2000). In addition, dung fuels can be re-used for several firing events and later stored for secondary uses such as manuring, all of which points to the value of dung-products and their potential sustainability in the present day (e.g. Portillo et al. 2017).

With regard to the use and recycling of dung and its by-products, in the Central Anatolian plateau Anderson and Ertuğ-Yaras (1998) reported that cattle dung was collected from enclosures and then dried and sieved to create a residue locally called *kön*, which is a dung by-product used as bedding for pens as well as fertilizer. At Neolithic Çatalhöyük, high stable isotope nitrogen values in crop plants indicate that livestock dung was used to fertilize cultivated plots and maintain soil fertility (Bogaard 2005). Fuel rake-outs with some dung inclusions were used in building materials such as mortars, possibly due to the hygro-phobic properties of ash (Matthews et al. 1996; Matthews 2010), and in mudbricks, introduced either deliberately or accidentally during the manufacturing process, as reported ethnographically (Matthews 2010; Love 2012; Portillo et al. 2014). In addition, the frequent deposition of fuel refuse composed of calcitic plant and dung ashes within open spaces has been suggested to relate to sanitation practices to reduce odours and insect breeding (Pawłowska 2014). In buildings, dung matter has only been rarely documented as part of floor construction materials in thin-section, mainly in the form of amorphous aggregates, randomly dispersed and less than a millimetre in size. The nature, frequency, distribution and size of these dung aggregates questions the intentional use of this material as stabilizers, pointing towards its accidental transfer into the plasters at the place of manufacture, likely middens and open spaces, where occupation refuse accumulated (García-Suárez 2017).

At the Late Neolithic campsite of Pınarbaşı open spaces have also been found to display accumulations of herbivore dung deposits interpreted to derive from repeated dung-burning events (García-Suárez et al. 2018). These were composed of high quantities of well-preserved dung spherulites and phytoliths from wild grasses, in addition to leaves and culms interpreted as the remains of fodder and fuel sources. The occurrence of charred and partially calcined faecal matter strongly associated with calcitic ashes and darkened spherulites in thin-section points to the extensive use of dung as fuel at seventh millennium Pınarbaşı, along with other woody sources such as *Pistacia* and *Amygdalus*, which were likely available in the vicinity according to the charred wood datasets (Asouti 2003). The good preservation conditions of these microfossils, and particularly dung spherulites, that are susceptible to dissolution when organic matter degrades in acidic burial environments and under calcination (Canti 1999), have been related to low/moderate firing temperatures as well as to rapid disposal and/or burial of used fuel at the site (García-Suárez et al. 2018).

Overall, the reported case-studies illustrate the vital role of livestock as providers of a range of dung-products and their sustainable applications, such as a fuel source, in this key region through time. Therefore, dung as an archaeological material deserves to be considered in syntheses on the emergence and spread of Neolithic innovations including the secondary products revolution (Sherratt 1983) more widely.

## Conclusions

This review illustrates the strength of interdisciplinary studies of dung as a highly valuable archaeological material, especially if conducted in comparison to modern reference datasets from the same research area using an ethnoarchaeological approach. The integration of micromorphology, plant and faecal microfossils, and biomolecular analyses, combined with reference ethnoarchaeological approaches, has provided a powerful suite of evidence for identifying and understanding the archaeological significance of livestock dung and other coprolitic materials within agricultural built environments across territories in Central Anatolia during the early Holocene. This review also highlights the value of comparative ethnoarchaeological datasets in providing models on factors concerning animal dung characteristics and uses, as well as the natural and anthropogenic pathways affecting their deposition and preservation. In future research, a range of issues remains to be addressed more systematically, including use of dung and dung-contents as indicators for paleoenvironment and seasonality, and of dung markers for exploring both livestock and human health and life conditions.

Dung remains have been found to represent important archaeological materials for investigating human-animal inter-relations and the developments of early farming communities of the early Holocene Anatolian plateau. These have provided important insights into continuity and change in herd management strategies and early husbandry, ecological diversity and foddering/grazing practices, showing a significant variation in livestock diet, including direct evidence from both grass-based and dicotyledonous regimes from recently discovered penning areas at early Neolithic Çatalhöyük (Matthews and Portillo 2017; Portillo et al. 2019). The nature of animal dung deposited and accumulated in open areas appears to be directly associated with the scale of pastoral activities at the sites investigated. At the early agricultural settlement of Boncuklu, where isotopic data on faunal bones suggest possible experimentation with caprine herd management (Middleton 2018; Baird et al. 2018), dung remains have been identified through micromorphology within the fills of open hearths, attesting the presence of ruminants near the site (García-Suárez 2017; García-Suárez et al. 2018). The substantial penning deposits identified at the early occupation of the long-lived site of Çatalhöyük (Matthews 2005a, b; Portillo et al. 2019) indicate the occurrence of pastoral activities within the settlement. However, micro-stratigraphic data point to a decline in the accumulation of dung matter within the settlement corresponding to a period of site expansion, and a later period of increased human and animal mobility (García-Suárez et al. 2018; Henton 2012, 2013; Pearson et al. 2015). By contrast, the seventh millennium pastoral campsite of Pınarbaşı, contemporary at this time with the late occupation of Çatalhöyük, displays abundant accumulations of re-deposited herbivore dung, most likely as a result of the use of this rockshelter as a herding location (Baird et al. 2011; García-Suárez et al. 2018).

Overall, integrated micro-contextual approaches reveal considerable chronological and contextual variation in human-animal inter-relations within and between these communities through time. Nonetheless, common patterns pointing to shared practices emerge from these data, such as the proximity of domestic animals and the use of dung as a secondary product (e.g. Matthews 2005a; Rosen 2005; Ryan 2011; Shillito 2011; García-Suárez et al. 2018). The use of dung fuel sources has been reported in all investigated sites, particularly in open contexts, a practice which is still in use among local communities in the present day. However, the intentionality of this practice at the early site of Boncuklu still needs to be explored further, due to the low amounts of this material identified so far in fire contexts.

Furthermore, this paper shows the ways in which high-precision identification and interpretation of dung remains can be used to address key archaeological questions in current debates in the Neolithic, not only on the origins and spread of farming, but also regarding the use and organization of space, patterns of co-habitation of humans with animals and livestock dung, as well as well-being and health challenges associated with early farming and food production. Animal penning in the earlier occupation of Çatalhöyük represents an increased exposure to pathogens and spread of diseases in living areas, with critical implications for health conditions of early settled life. As such, the contribution of this important archaeological material, deserves to be more systematically approached and fully considered in syntheses on the emergence and spread of early farming systems, particularly since the domestication of herds, and its role in the secondary products revolution.
